# Molecular Characteristics of Methicillin-Resistant *Staphylococcus aureus* (MRSA) Isolated from Diabetic Foot Infection

**DOI:** 10.30699/ijp.2019.101092.2035

**Published:** 2019-09-22

**Authors:** Pegah Kananizadeh, Solmaz Ohadian Moghadam, Yasaman Sadeghi, Abbas Rahimi Foroushani, Hossein Adibi, Mohammad Reza Pourmand

**Affiliations:** 1 *Department of Pathobiology, School of Public Health, Tehran University of Medical Sciences, Tehran, Iran*; 2 *Uro-Oncology Research Center, Tehran University of Medical Sciences, Tehran, Iran*; 3 *Department of Epidemiology and Biostatistics, School of Public Health, Tehran University of Medical Sciences, Tehran, Iran*; 4 *Diabetes Research Center, Endocrinology and Metabolism Clinical Sciences Institute, Tehran University of Medical Sciences, Tehran, Iran *

**Keywords:** Diabetic foot infection, Methicillin-resistant Staphylococcus aureus, SCCmec typing

## Abstract

**Background & Objective:**

Diabetic foot ulcer (DFU), is one of the most frequent causes for hospitalizations in patients with diabetes. A major problem in the treatment of DFU is the increased-incidence of methicillin-resistant *Staphylococcus aureus *(MRSA). The aim of this study was to determine the SCC*mec* types of MRSA isolates and their epidemiology among patients with diabetes.

**Methods:**

This study was carried out on 145 diabetic patients with DFUs. The antibiotic susceptibility tests (ASTs) were performed using the disk diffusion method and E-test technique. SCC*mec* typing was done by multiplex PCR. Moreover, the presence of virulence toxin genes, including *pvl* and *lukED* was detected by PCR assay.

**Results:**

In 145 samples from which *S. aureus* was predominantly isolated, 19.48% were MRSA. Analysis of MRSA isolates revealed that the most prevalent SCC*mec* type was type IV (46.7%) followed by type III (30.0%) and type V (20.0%). One strain (3.3%) was untypeable. The prevalence of *pvl* and *lukED* was 56.7% and 100%, respectively.

**Conclusion:**

The high prevalence of MRSA in DFUs represents the high levels of antibiotic usage among patients with diabetes. In this study, resistance to other important clinical antibiotics was detected among MRSA isolates. The high proportion of SCCmec type IV and V strains, even in former hospitalized patients, indicates the entrance of these clones to the clinical setting.

## Introduction

Diabetes mellitus (DM), as one of the four priority non-communicable diseases, is an expanding global health problem and an important reason for premature death and disability. Diabetes, as a serious chronic disease, has several complications such as diabetic foot ulcers (DFUs) (1), and infection of these ulcers is a common (40%–80%) complication that leads to the hospitalization of a patient (2). 

Methicillin resistant *Staphylococcus aureus* (MRSA) strains play a significant role as an important pathogen in diabetic foot infection (DFI) and have become a public health concern due to their increased virulence and resistance to an increasingly broad spectrum of antibiotics (3). The two types of MRSA, including hospital-associated (HA) and community-associated (CA), can be differ-entiated according to staphylococcal chromosomal cassette *mec* (SCC*mec*) types. SCC*mec* elements in MRSA are classified into different types based on the combination of *mec* and *ccr* gene complexes (4). Most of HA-MRSA harbor SCC*mec* I-III types, but more SCC*mec* IV and V types are present in CA-MRSA. In addition, a much lower resistance to antibiotics is seen among CA-MRSA than in HA-MRSA. Therefore, the SCC*mec* typing was done as a functional molecular tool to clarify the various structures of SCC*mec* elements and to understand the essential aspect of the epidemiology of MRSA (4).

Due to the expression of various virulence factors, including pore-forming toxins such as Panton–Valentine leukocidin (PVL) and leukotoxins (luk-ED) which confer leukocyte destruction and tissue necrosis, CA-MRSA strains are considered more virulent than HA-MRSA. The *pvl* genes are associated with more severe invasive diseases and poor prognosis, and are more likely to be isolated from community rather than hospital settings (5). With regards to the high incidence of HA- and CA-MRSA strains in DFUs, genomic characterization of MRSA isolates may improve our knowledge about molecular epidemiology of this pathogen. The aim of this study was to determine the SCC*mec* types of MRSA isolates among patients with diabetes. 

## Materials and Methods

**Study Design and Bacterial Isolates**

In this cross-sectional study, a total of 145 specimens including pus, exudates from lesions, and tissue biopsies were obtained during January 2017 to August 2017 from patients with Diabetic foot infection in the Diabetes Research Center, Endocrinology and Metabolism Clinical Sciences institute, Tehran, Iran. Samples were acquired by sterile swabbing from the ulcer base and tissue biopsies were obtained by scraping the ulcer with a sterile curette. Then *S. aureus* isolates were identified by standard biochemical and microbiological techniques (Gram’s stain, catalase, coagulase and DN*ase* activities and mannitol fermentation on mannitol salt agar).

All participants gave written informed consent. The characterization and severity of DFU were assessed based on the Wagner Ulcer Classification System, which classified all DFUs in five major categories including a superficial diabetic ulcer, ulcer extension, deep ulcer with abscess or osteomyelitis, gangrene to a portion of forefoot and extensive gangrene of foot (6).

A clinico-demographic questionnaire was arranged for data collection. The questions were pertained to demographic information for each patient including age, sex, prior antibiotic usage (≤ 3 months), prior hospitalization, implantation of percutaneous medical device, experience of any surgery or prior residence in a long-term healthcare facility within the 6 months prior to the sampling date, the course of DM, the course of the ulcer, previous ulcers and amputation history, ulcer grade, HbAlC, lifestyle factors, presence of retinopathy, nephropathy, neuropathy, and peripheral vascular disease. Symptoms such as extensive gangrenous cellulitis in toes or in the rest of the foot, necrotizing fasciitis, sepsis, exudate formation and some amputation cases in which the infection persisted, were the inclusion criteria in this study.

**DNA Extraction**

Chromosomal DNA of MRSA strains were extracted using the Sinapure-DNA kit. Prior to DNA extraction, the isolates were sub-cultured in tryptic soy broth at 37°C for 24 h. Culture material were pelleted by centrifugation and were enzymatically lysed with resuspension in 100 μL prelysis buffer and 20 μL lyzosyme, and incubated for at least 30 min. At the lysis step, 10 μL ributinase was added and temperature was increased to 55°C. After that a 30 min incubation procedure was performed according to instructions. 

**Detection of MRSA**

Methicillin resistance was determined on Mueller–Hinton agar by using the 30 μg cefoxitin disk. *S. aureus* colony suspension, equivalent to 0.5 McFarland turbidity was inoculated on Mueller–Hinton agar and incubated at 33 to 35°C for 16 to 18 h. The interpretation of the results was performed according to Clinical and Laboratory Standards Institute (CLSI) guidelines. Resistance to methicillin was confirmed by the detection of the *mecA* gene by PCR.

**Scc** ***mec*** **Typing by Multiplex PCR:**

Purified genomic DNA was used as the template for SCC*mec* typing, based on *ccr* and *mec* gene complex typing, without determining the differences in the junkyard region. Multiplex PCR was carried out using primers suggested by Jarraud *et al.* (7). Our PCR products were sequenced and aligned based on SCC*mec* sequenced reference strains including: type III (AB037671), type IV (AB063173) and type V (AB121219) (4).

PCR amplification was performed in a volume of 25 μL with SinaClon PCR Master Mix 2X. The reaction mixtures consisted of: DNA template 2 μL, oligonucleotide primers (1-2 μL), 2× PCR buffer) 12.5 μL and H_2_O to get a final reaction volume of 25 μL. PCR amplification was performed using SENCOQUEST labcycler and was programmed for identifying *mec*A and *ccr* genes as follows: initial denaturation at 94°C for 2 min, 35 cycles of denaturation (94°C for 2 min), annealing (60°C for 1:30 min), extension (72°C for 2 min) and a final elongation at 72°C for 2 min. PCR amplification for identifying *mec* gene complex classes was programmed as follows: initial denaturation at 94°C for 1 min, 30 cycles of denaturation (94°C for 1 min), annealing (60°C for 1 min), extension (72°C for 2 min) and a final elongation at 72°C for 2 min.

SCC*mec* types that could not be categorized with a set of primers and under any of these types were classified as untypeable (4).

Our nucleotide sequences were submitted to GenBank, which in turn provided GenBank accession numbers for our nucleotide sequences:

BankIt2083483 SCC*mecA* MG874129 to BankIt 2083483 luk MG874136.

**Detection of Toxin Genes**

Sequences specific for *lukS*-PV–*lukF*-PV, *lukE*, *lukD* encoding *pvl* components S and F; *luk*E, *luk*D respectively, were detected by PCR SENCOQUEST lab cycler with primers suggested by Jarraud *et al*. (7). These primer sequences correspond to 433bp of the *pvl* gene and 269bp of *lukED* gene. We used *Staphylococcus* *aureus* (GenBank accession number Y13225), and *luk*SI and *luk*F-I of Staphylococcus intermedius (GenBank accession number X79188) as a positive control. PCR amplification was performed as previously described (8).

**Antibiotic Susceptibility Tests**

The antibiotic susceptibility tests (ASTs) were performed by using the Kirby–Bauer disk diffusion method on Muller–Hinton agar plates and the e-test technique was used to determine the minimal inhibitory concentration (MIC) of vancomycin according to Clinical and Laboratory Standards Institute (CLSI). Susceptibility tests for gentamicin, doxycycline, ciprofloxacin, erythromycin, clindamycin, trimethoprim-sulfamethoxazole, chloramphenicol, rifampicin, linezolid, teicoplanin, mupirocin and vancomycin were performed in accordance with the CLSI guideline 2016. *S. aureus* strain ATCC 25923 was used as a control for susceptibility testing.

The control strain was stored at -70℃ and cultured on nutrient agar at 4-8℃. Before testing, the strains were sub-cultured to agar plates. Any alteration in the mean zone diameters with control strains may be explained as a mutation or contamination. The zone diameters of our samples were within the standards of the control strain.

We also performed the standard control of antimicrobial discs for disc diffusion methods. Working stocks were kept below 8℃ β

*S. aureus* strain ATCC 25923 is commonly used as a control strain for susceptibility testing to antibiotics and as a quality control strain for commercial products.

**Statistical Analysis**

The results were analyzed by SPSS 23.0 (SPSS Inc., Chicago, IL., USA) using Fisher’s exact test, Pearson’s *X 2* test and independent sample t test, where appropriate (demographic information, virulence factors and AST results). A P-value of <0.05 was considered to be statistically significant.

## Results

**Characteristics of Patients Infected with MRSA and MSSA Strains**

**Fig. 1:**
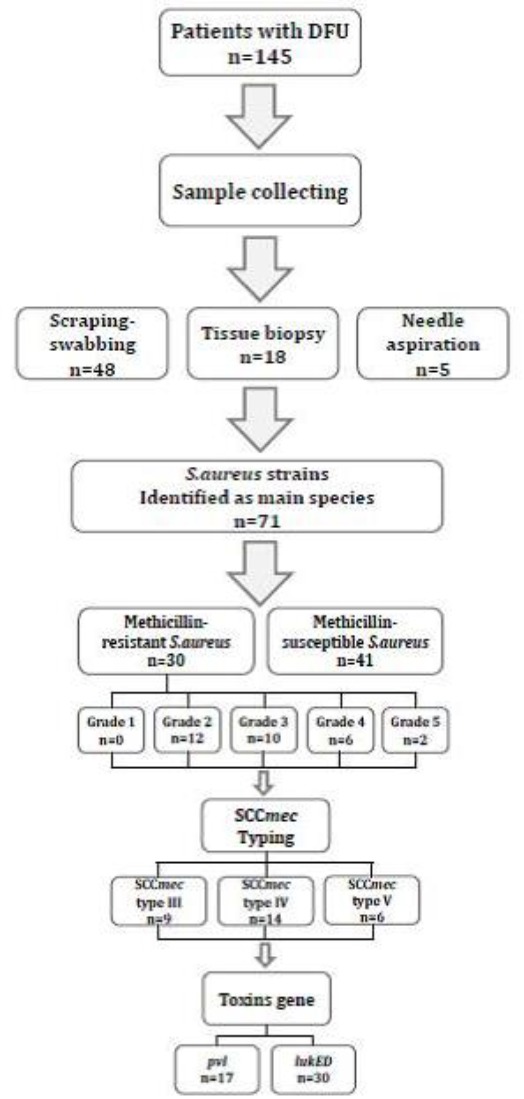
Flow of sample collection from patients with diabetic foot ulcer (DFU) through the study and methicillin-resistant detection of *S. aureus* strains. SCC*mec* types determination and toxins gene obtained on MRSA

In 145 samples from infected DFUs, 83 (53.89%) were predominately aerobic gram-positive organisms, of which 71 (46.10%) were identified as *S. aureus*. Different stages of the study procedure were exhibited in the flow-chart (Figure 1). The demographic and clinical characteristics of patients with DFUs infected with *S. aureus* isolates are shown in Table 1. Statistical analysis showed that antibiotic usage (*P*=0.043) and prior hospitalization (*P*=0.003) had significantly affected MRSA isolation from diabetic patients. More than two-thirds of the patients with MRSA infections (25 from 30) had undergone recent antibiotic therapy (≤3 months), while patients with MSSA infections were currently undergoing antibiotic therapy, or did not undergo any antibiotic therapy at all.

**Table 1:** Characteristics and demographic data of patients with DFUs infected with MRSA and MSSA strains

Characteristic	Patient with MSRA	Patient with MSSA	Total	P-value
	n=30	n=41	n=71	MSSA vs. MRSA
**Age (years)**	62.43(40-77)	60.29(36-85)	61.19(36-85)	0.392
**Sex n (%)**				
**Male/Female**	22(73.3)/8(26.7)	30(73.2)/11(26.8)	52(73.2)/19(26.8)	0.988
**The course of DM (years)**	15.93(3-40)	14.04(3-37)	14.84(3-40)	0.375
**HbA1c**	8.17±1.3	7.69±1.3	7.89±1.3	0.152
**Cardiovascular Disease**				
**Arterial Hypertension**	18(60.0)	28(68.3)	46(64.8)	0.47
**Hyperlipidemia**	16(53.3)	28(68.3)	44(62.0)	0.2
**Peripheral Arterial Disease**	12(40.0)	22(53.7)	34(47.9)	0.254
**Coronary Heart Disease**	10(33.3)	21(51.2)	31(43.7)	0.153
**Nephropathy**				
**Micro-albuminuria**	14(46.7)	17(41.5)	31(43.7)	0.662
**Macro-albuminuria**	11(36.7)	13(31.7)	24(33.8)	0.663
**ESRD**	11(36.7)	13(31.7)	24(33.8)	0.665
**Peripheral Neuropathy**				0.046
**Mild**	1(3.3)	3(7.3)	4(5.6)	
**Middle**	9(30.0)	21(51.2)	30(42.3)	
**Sever**	20(66.7)	17(41.5)	37(52.1)	
**Retinopathy**				
**Cataracts**	15(50.0)	19(46.3)	34(47.9)	0.762
**Glaucoma**	4(13.3)	3(7.3)	7(9.9)	0.446
**Lifestyle Factors**				
**Obesity**	16(53.3)	16(39)	32(45.1)	0.231
**Smoking**	10(33.3)	11(26.8)	21(29.6)	0.553
**Alcoholism**	3(10.0)	4(9.8)	7(9.9)	1
**Activity**	10(33.3)	22(53.6)	32(45.1)	0.109
**Drugs**	6(20.0)	8(19.5)	14(19.7)	0.959
**The Course of Ulcer (days)**	92.26(1-365)	81.39(1-365)	85.98(1-365)	0.725
**Previous Ulcer /Amputation**	21(70.0)/9(30.0)	26(63.4)/5(12.2)	47(66.2)/14(19.7)	0.565/0.080
**Wagner's Grades**				0.066
**1**	-	2(4.9)	2(2.8)	
**2**	12(40.0)	20(48.8)	32(45.1)	
**3**	10(33.3)	16(39.0)	26(36.6)	
**4**	6(20.0)	1(2.4)	7(9.9)	
**5**	2(6.7)	2(4.9)	4(5.6)	
**Antibiotic use**	25(83.3)	25(61.0)	50(70.4)	0.043
**Recent Hospitalization**	16(53.3)	8(19.5)	24(33.8)	0.003
**Samples**				0.049
**Scraping-swabbing**	16(53.3)	32(78.0)	48(67.6)	
**Tissue Biopsy**	12(40.0)	6(14.6)	18(25.4)	
**Needle Aspiration**	2(6.7)	3(7.3)	5(7.0)	

**Virulence Profiles in Patients Infected with MRSA**

**Fig. 2:**
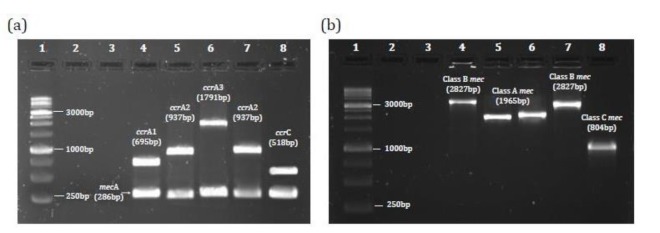
PCR products of SCC*mec* typing (agarose 1%): (a) Lane 1: MSM (1kb molecular size marker). Lane 2: Non-Template Control (NTC). Lane 3: Negative Control of *mecA* (MSSA). Lane 4: *ccr*A1 of *ccr* gene complex- Type I SCC*mec* (control strain). Lane 5: *ccr*A2 of *ccr* gene complex- Type II SCC*mec* (control strain). Lane 6: *ccr*A3 of *ccr* gene complex- Type III SCC*mec* (positive specimen of diabetic isolates). Lane 7: ccrA2 of *ccr* gene complex- Type IV SCC*mec* (positive specimen of diabetic isolates). Lane 8: *ccr*C of *ccr* gene complex- type V SCC*mec* (positive specimen of diabetic isolates). (b) Lane 1: MSM (1kb molecular size marker). Lane 2: Non-Template Control (NTC). Lane 3: Negeative Control of *mec*A (MSSA). Lane 4: class B *mec* gene complex- Type I SCC*mec* (control strain). Lane 5: class A *mec* gene complex - Type II SCC*mec*. Lane 6: class A *mec* gene complex - Type III SCC*mec*. Lane 7: class B *mec* gene complex- Type IV SCC*mec*. Lane 8: class C *mec* gene complex- Type V SCC*mec*

Using PCR method for the detection of *mecA* gene in 71 *S. aureus* isolated from DFUs, 30 (42.25%) isolates were confirmed as MRSA and 41(57.74%) isolates were methicillin susceptible. Based on the SCC*mec* typing by multiplex-PCR assay, 14 (46.7%) strains belonged to SCC*mec* type IV, 9 (30.0%) strains to SCC*mec* type III, and 6 (20.0%) to SCC*mec* type V. One isolate (3.3%) was classified as untypeable and did not belong to any of the SCC*mec* types (Figures 2a and 2b). 

The* pvl* and *lukED* genes encoding the bicomponent leukotoxin (LukS-LukF and LukE-LukD, respectively), were detected by PCR. Results indicated that 17 (56.7%) strains of MRSA isolates harbored* pvl* gene, 15 (88.2%) of which included CA-MRSA (*P*=0.023). Genes encoding for LukED were found in all 30 (100%) MRSA isolates. The *pvl* gene was often detected in strains isolated from ulcer grade 2 and 3 (*P*=0.026), whereas the *lukED* gene was found in all strains from ulcer grade 2-5. Statistical analyses revealed that PVL produced by MRSA does not associate with factors such as previous ulcer, ulcer duration, prior hospitalization, record of amputation, and antibiotic usage (Table 2)

.

**Table 2:** Molecular characteristics of MRSA isolates recovered from DFU infections

characteristics		PVL-Positive isolates	PVL-negative isolates	P-value	*luk ED*- positive isolates
		n=17	n=13		n=30
**Wagner's grade**				0.026	
**Grade 1 (n=0)**		0 (0)	0 (0)		0 (0)
**Grade 2 (n=12)**		9 (52.9)	3 (23.1)		12(40)
**Grade 3 (n=10)**		6 (35.3)	4 (30.8)		10(33.3)
**Grade 4 (n=6)**		2 (11.8)	4 (30.8)		6(20)
**Grade 5 (n=2)**		0 (0)	2 (15.4)		2(6.6)
SCCmec types*				0.023	
**III (n=9)**		2 (11.8)	7 (58.3)		9(31.03)
**IV (n=14)**		11 (64.7)	3 (25)		14(48.27)
**V (n=6)**		4 (23.5)	2 (16.7)		6(20.68)
**Antibiotic usage**		15( 88.2)	10 (76.9)	0.628	25(83.3)
**Recent hospitalization**	6 (35.3)	10 (76.9)	0.033	16(53.3)
**previous amputation (n=9)**		3 (17.6)	6 (46.2)	0.128	9(30)
**previous ulcer (n=21)**		12(40)	9(30)	0.567	21(70)
**The course of ulcer (days)**		109.29±116.574	70±93.015	0.592	92.2±6107.09

* one strain was classified as untypeable.

**Antimicrobial Susceptibility Patterns in MRSA Strains**

All MRSA strains were sensitive to linezolid, mupirocin and vancomycin. The highest resistance rate (100%) among the HA-MRSA isolates was seen for gentamicin, ciprofloxacin, erythromycin, clindamycin, and rifampin. However, the CA-MRSA isolates were more susceptible to gentamicin (100%), rifampin (90%), ciprofloxacin (60%) and doxycycline (55.0%). The sensitivity to doxycycline (*P*=0.013), ciprofloxacin (*P*=0.002) and rifampin (*P*<0.001) antibiotics was statistically significant between HA-MRSA and CA-MRSA. Both HA- and CA-MRSA isolates showed the same susceptibility pattern to the other study antibiotics. No vancomycin-resistant strain was found among MRSA isolates (Table 3).

**Table 3:** Antibiotic susceptibility pattern of HA-MRSA and CA-MRSA strains

Antimicrobial agents n(%)	CA-MRSA=20			HA-MRSA=9			P-value
	S	I	R	S	I	R	
**Cefoxitin**			20(100)			9(100)	-
**Gentamicin**	20(100)					9(100)	-
**Doxycycline**	11(55.9)	4(20.0)	5(25.0)		4(44.44)	5(55.55)	0.013
**Ciprofloxacin**	12(60.0)	1(5.0)	7(35.0)			9(100)	0.002
**Erythromycin**	1(5.0)	2(10.0)	17(85.0)			9(100)	0.458
**Clindamycin**	1(5.9)	3(15.0)	16(80.0)			9(100)	0.259
**Trimethoprim-sulfamethoxazole**	18(90.0)		2(10.0)	9(100)			1
**Chloramphenicol**	13(65.0)	7(35.0)		5(55.55)	4(44.44)		0.694
**Rifampin**	18(90.0)		2(10)			9(100)	<0.001
**Linezolid**	20(100)			9(100)			-
**Mupirocin**	20(100)			9(100)			-
**Teicoplanin**	12(60.0)	8(40.0)		4(44.44)	5(55.55)		0.688
**Vancomycin**	20(100)			9(100)			-

## Discussion

The results of SCC*mec* typing on MRSA strains isolated from diabetic ulcers showed that most strains (66.7%) harbor SCC*mec* types IV and V, which belong to CA-MRSA. Since both strains are separated from both sources, it is logical to use the word “associated” instead of acquired. This can be truer for CA-MRSA, which recently entered from its original site into the hospital setting with the ability to develop a nosocomial infection, especially skin and soft tissue infection (9).

One of the main differences between CA- and HA- MRSA is resistance to different types of antibiotics, except beta-lactams. The initial hypothesis about strains with SCC*mec* types IV and V was that they had much lower resistance to antibiotics than strains with SCC*mec* types I, II and III (10). Antibiotic susceptibility patterns also revealed that isolates classified as SCC*mec* type III were resistant to more than three antibiotic classes, while isolates with SCC*mec* types IV and V showed even more susceptibility. Even though CA-MRSA strains were more sensitive than HA-MRSA strains, a high resistance of more than 50% to two antibiotic classes was observed. Due to the fact that CA-MRSA can be found in hospital settings, it is believed that CA-MRSA strains in hospitals show higher resistance to non-β-lactam antibiotics (11). In this investigation also, seven (23.33%) MRSA strains, which were isolated from hospitalized patients, were CA-MRSA based on SCC*mec* type. Obtained results showed that these strains, which harbor SCC*mec* types IV and V, have similar antibiotic susceptibility patterns to MRSA strains classified as SCC*mec* type III. The lower rate of susceptibility to the doxycycline and ciprofloxacin strains was seen in CA-MRSA strains in hospital, compared with CA-MRSA strains in community. According to previous study, the susceptibility rates among HA-SCC*mec*-IV isolates was significantly less for clindamycin, gentamicin, and levofloxacin, compared with SCC*mec*-IV isolates acquired in the community (9). 

In Iran, antimicrobial therapy of DFIs is done according to the ulcer’s grade and the severity of infection, which is based on the global Empiric Antibiotic Regimens for Diabetic Foot Ulcers (6). Since more patients were diagnosed with moderate and severe infections, ciprofloxacin was the most prescribed antibiotic. The statistical connection was seen between patients with MRSA infections, who had received antibiotics in the recent three months and previous fluoroquinolone therapy (ciprofloxacin (*P*=0.002). This result was also in accordance with a study done by Mendes et al. (12). According to the recent study, this class of antibiotics correlates with the spread of multi-drug resistant organisms. MRSA in particular could be a potential cause (12). With 100% sensitivity among all isolates, linezolid, mupirocin and vancomycin are considered the most effective antibiotics against MRSA strains with no difference in the origin of bacterial strains. In addition, erythromycin, with 10% sensitivity among all isolates, was the least effective one. 

In this study, *S. aureus* (46.1%) was the most commonly isolated bacteria from infectious diabetic ulcer, which is consistent with other studies (13, 14). The prevalence of 42.2% methicillin-resistance was observed among *S. aureus* strains. DFU infection, as a major complication of diabetes, results in a higher risk of lower extremity amputation and hospitalization (1). Reports during the past 10 years of studies indicated that MRSA has emerged as a serious problem in DFUs because of changes in MRSA epidemiology and the growing rate of infections caused by MRSA (15).

In the current study, most patients with DFUs were male (64.9%). According to a previous study related to the prevalence of DM, females were more likely to have DM than males (16), while male gender is more likely to have DFU than female. We also found that the number of the MRSA isolated was remarkably higher in men than in women [22(73.3%) vs 8(26.7%)], which is consistent with other reported studies (17, 18). 

In obtained outcomes, almost half of the patients with MRSA infections had a history of hospitalization. Hospital stay could be due to a high prevalence of diabetic complications (19). We noticed that the greater prevalence of infections due to MRSA was significantly seen in patients with recent antibiotic therapy, mainly hospitalized ones. Data analysis showed that the average duration of an ulcer with MRSA infection was 92.26 days. We concluded that MRSA isolation from patients with diabetic ulcer did not significantly affect the ulcer’s persistence. Furthermore, another study indicated that among patients who had a multidrug resistant organism (mostly MRSA), the causative pathogen was not associated with duration of wound healing (20). We observed that infection with MRSA could affect the amputation in diabetic patients, but this was not statistically significant. While other retrospective studies of DFI have found infection with MRSA associates with ulcer persistence, as well as, higher amputation risk (21).

Various studies have investigated different methods of sampling from DFUs. It is believed that superficial swab cultures of DFIs may contain colonized skin organisms, rather than the causative agent of the infection. While tissue biopsies and fluid aspirates are considered more accurate than swabbing, it has been reported that the use of a wound swab after debridement is as reliable as the use of a tissue specimen (22). Sampling with invasive techniques is not used frequently in practice settings, such as outpatient clinics, due to the worsening of the wound. Therefore, with regards to this limitation in the present study, the standard swab protocol was used for sampling, with sufficient precision to prevent surface contamination. In current results, there was a significant difference between two types of sampling. Rate of MRSA isolation from tissue biopsy samples were significantly high, while this was not significant in swab and aspirates samples.

Many studies have shown that CA-MRSAs are increasingly isolated in Skin and soft tissue infections, so it can be one of the most important pathogens in diabetic ulcers (23). Many CA-MRSA strains that harbor either SCC*mec* type IV or SCC*mec* type V elements produce PVL (24). Although this toxin is a causative agent of severe tissue necrosis due to cytotoxicity activity on PMNs, in this project 15 (88.2%) *pvl*- positive strains were isolated from ulcer grade 2 and 3, while only 2 (11.8%) of them were from ulcer grade 4 and 5. According to other studies also, toxin-producing strains are rarely isolated from DFUs, as a chronic wound (25). The results showed that the most *pvl* positive strains were isolated from grade 2 ulcers; however, the detection of *pvl* positive strains was associated with wound chronicity (*P*=0.026). Gradually, as the wound-healing prolonged, the chronicity increased and the isolation of *pvl *positive strains decreased. Four out of seven HA- SCC*mec* type IV and V strains were also *pvl *positive.

The *lukED* gene was detected in all strains and was equally distributed among 2-5 ulcers grades. *lukED* gene, which was found in about 85% of the *S. aureus* strains, is encoded by a stable pathogenicity island. The detection of *lukED* in MRSA strains has been also reported in DFI (20). The cytotoxic activity of LukED is induced in the in vivo pro-inflammatory response by targeting specific immune cells (8). However, according to a study conducted by Shu-Hong Feng (3), LukED presents poorer cytotoxic effects in comparison with PVL. The reduced virulence and inflammatory factors related to LukED cause an atypical local inflammatory reaction among MRSA-infected patients.

In this study, the high incidence of MRSA was seen between DFIs. ASTs also revealed the high potential of these pathogens in presenting resistance to other important clinical antibiotics. Furthermore, the high prevalence of strains with SCC*mec *types IV and V indicates the recent emergence of these strains in healthcare settings, which leads to a higher possibility of nosocomial infections. This project will provide a warning to experts and policymakers in this field to improve prevention and control programs and treatment in DFIs in order to reduce resistance patterns, and healthcare costs.
